# Selection Processing in Noun and Verb Production in Left- and Right-Sided Parkinson's Disease Patients

**DOI:** 10.3389/fpsyg.2018.01241

**Published:** 2018-07-20

**Authors:** Sonia Di Tella, Francesca Baglio, Monia Cabinio, Raffaello Nemni, Daniela Traficante, Maria C. Silveri

**Affiliations:** ^1^IRCCS, Fondazione don Carlo Gnocchi ONLUS, Milan, Italy; ^2^Department of Pathophysiology and Transplantation, Università degli Studi di Milano, Milan, Italy; ^3^Department of Psychology, Università Cattolica del Sacro Cuore, Milan, Italy

**Keywords:** Parkinson's disease (PD), executive functions, word production, magnetic resonance imaging, Inferior Frontal Gyrus (IFG), Broca's area, brain thickness

## Abstract

Verbs are more difficult to produce than nouns. Thus, if executive resources are reduced as in Parkinson's disease (PD), verbs are penalized compared to nouns. However, in an experimental condition in which it is the noun that must be selected from a larger number of alternatives compared to the verb, it is the noun production that becomes slower and more prone to errors. Indeed, patients are slower and less accurate than normal subjects when required to produce nouns from verbs (VN) in a morphology derivation tasks (e.g., “osservazione” from “osservare”) [“observation” from “observe”] than verbs from nouns in a morphology generation task, in which only a verb can be generated from the noun (NV) (e.g., “fallire” from “fallimento”) [“to fail” from “failure”]. In the Italian language morphology, in fact, generation and derivation tasks differ in the number of lexical entries among which the response must be selected. The left Inferior Frontal Gyrus (IFG) has been demonstrated to be involved in selection processes. In the present study, we explored if the ability to select words is related to the cortical thickness of the left IFG. Twelve right-sided PD with nigrostriatal hypofunctionality in the left hemisphere (RPD-LH), 9 left-sided PD with nigrostriatal hypofunctionality in the right hemisphere (LPD-RH) and 19 healthy controls (HC) took part in the study. NV and VN production tasks were administered; accuracy and reaction times (RTs) were collected. All 40 subjects received a structural MRI examination. Cortical thickness of the IFG and volumetric measurements for subcortical regions, thought to support selection processes, were computed using FreeSurfer. In VN derivation tasks RPD-LH patients were less accurate than LPD-RH patients (accuracy: 66% vs. 77%). No difference emerged among the three groups in RTs. Task accuracy/RTs and IFG thickness showed a significant correlation only in RPD-LH. Not only nouns (as expected) but also verbs were correlated with cortical thickness. This suggests that the linguistic nature of the stimuli along with executive resources are both relevant during word selection processes. Our data confirm that executive resources and language interact in the left IFG in word production tasks.

## Introduction

The disproportionate impairment in the production of verbs compared to nouns, frequently documented in patients with Parkinson's disease (PD) (Silveri et al., 2018), can be considered an expression of the dysexecutive syndrome, the typical manifestation of PD patient's cognitive decline (Dubois and Pillon, 1996; Roussel et al., 2017). Deficit for verbs in a pathology typically dominated by disorders of movement has been also attributed to the decay of conceptual representation of the motor components of the action (Boulenger et al., 2008; Rodríguez-Ferreiro et al., 2009; Cardona et al., 2014), according to an embodied view of cognition (Barsalou, 1999).

For a series of reasons, verbs are, among the grammatical classes, more difficult to produce than nouns (Mätzig et al., 2009). Thus, in the presence of reduced executive resources (such as in extrapyramidal pathologies), the verb class is more penalized than the noun class (Cotelli et al., 2006; Silveri et al., 2012). For example, during word production, verb forms must be selected from a larger set of word forms with the same verb root compared to nouns. Consequently, the selection process requires the recruitment of a larger amount of attentional resources. However, in an experimental condition in which it is the noun that must be selected from a larger number of alternatives compared to verb, noun production becomes slower and more prone to errors, thus more difficult. Indeed, in Silveri et al.'s study (2018) PD patients were slower and less accurate than healthy controls (HC) when required to produce nouns from verbs in a morphology derivation tasks (e.g., “osservazione” from “osservare”) [“observation” from “to observe”] than verbs from nouns in a morphology generation task, in which the verb must be generated from the noun (e.g., “fallire” from “fallimento”) [“to fail” from “failure”]. In the Italian language morphology, in fact, generation and derivation tasks differ in the number of lexical entries among which the response must be selected (see also Marangolo et al., 2006). For example, when the noun “cammino” [walk] must be produced (derived) from the verb “camminare” [to walk], the choice is from six alternative nouns (“cammino” [walk], “camminata” [walk], “camminamento” [route], camminante [walking], camminatrice [walker, female], camminatore [walker, male]); but, when the verb base “camminare” [to walk] must be produced (generated) from the derived noun “cammino” [walk], there is virtually only one choice. In addition, in the above cited study, frequency of target competitors and number of competitors more frequent than the target were also related to response latency and accuracy in word production tasks. These findings suggest that not only the selection process but also controlled retrieval is impaired in PD, as proposed by Crescentini et al. (2008).

Specifically, in the previous study (Silveri et al., 2018) PD patients were impaired not only when they had to select the target from many competitors, but also in the presence of competitors more frequent than the target. An efficient control of the lexical retrieval should inhibit their production, but inhibitory processes are impaired in PD (Castner et al., 2008) and competitors more frequent than the target were produced in the place of the target.

Results such as the ones above described would be consistent not only with a lack of inhibition of the irrelevant information represented by the competitors, but also with lack of top-down potentiation of the relevant information, potentiation supported by the basal ganglia (Norman and Shallice, 1986; Egner and Hirsch, 2005). Evidence from PD suggest that the basal ganglia are mostly active in post-retrieval processes, such as the inhibition of task irrelevant information (Crescentini et al., 2010), and also in the top down potentiation of relevant information (Norman and Shallice, 1986; Egner and Hirsch, 2005).

Moreover, neuroimaging studies in normal subjects have suggested that the left Inferior Frontal Gyrus (IFG) (or Ventrolateral Prefrontal Cortex-VLPC) is crucial in the selection processes (Moss et al., 2005; Siri et al., 2008) and that the IFG of the left hemisphere (LH) is involved in both selection processes and controlled retrieval (Badre et al., 2005; Moss et al., 2005; Thompson-Schill and Botvinick, 2006). Selection process and controlled retrieval are dissociable processes and, as such, potentially supported by different neural substrates within the IFG (Badre et al., 2005). For example, the IFG pars triangularis (Brodmann area-BA 45) supports the former whereas the anterior portion-pars orbitalis (BA 47) the latter. Consequently, the experimental evidence from both normal subjects and PD converges in suggesting that selection processes are supported by neural substrates that include both the IFG and subcortical regions (Mostofsky and Simmonds, 2008). IFG and the dorsal striatum (caudate nucleus and putamen) are included within the corticostriatal pathway, a key circuit associated with executive deficits in nondemented PD and motor inhibition (Owen, 2004; Baglio et al., 2011). The lack of inhibition is a characterizing aspect of PD dysexecutive syndrome (Obeso et al., 2011) which reaches the most striking clinical evidence in impulsive behavior (Napier et al., 2015). According to literature data, not only the VLPC (BA 45 and 47) but also the mid-dorsolateral frontal cortex (BA 46 and 9) are linked with different aspects of executive processing, and are crucial to optimize performance in a variety of executive tasks (Alexander et al., 1986; Owen, 2004).

Idiopathic PD generally presents, at initial phase, as an asymmetric clinical syndrome with right or left predominance, that correlates with neuronal loss in the deep gray matter of the contralateral hemisphere (Kempster et al., 1989; Lee et al., 2014; Tanner et al., 2017), although not systematically (Nemmi et al., 2015). Only a few studies looked specifically for the existence of structural cortical asymmetries in PD, suggesting that atrophy could start on one side (Zarei et al., 2013), and then extend to the other (Pereira et al., 2014; Tanner et al., 2017). Although the evidence of structural asymmetries extending to the cortical regions is for now weak, right-sided (RPD) and left-sided PD (LPD) are thought to have different cognitive profiles, with the former presenting dysfunction in language and verbal memory and the latter in spatial attention (see Verreyt et al., 2011 for a review). Thus, in general, clinical and experimental evidence suggests that symptom side predominance may predict, at least to some extent, decline in specific cognitive domains subtended by neural substrates in the contralateral hemisphere, although the involvement of the cortical areas, in addition to the subcortical regions, has not been incontrovertibly demonstrated. However, correlations between the structural indexes of specific brain regions and neuropsychological performances have been demonstrated in PD: executive abilities are correlated to the cortical thickness of frontal or frontoparietal regions (Pereira et al., 2009; Biundo et al., 2011; Filoteo et al., 2014; Duncan et al., 2016) and memory performance with the volume of hippocampus (Beyer et al., 2013).

In the present study we explored if disorders of the executive abilities, such as word selection among competing alternatives, could be subtended by changes in cortical thickness in the inferior regions of the frontal lobes; in particular, if changes in the LH could be detected when tasks of linguistic nature, such as word production tasks, are used. The role of the frontostriatal pathway of the LH in word selection processes is quite robust (Moss et al., 2005; Siri et al., 2008) whereas evidence of cortical changes in the left frontal cortex (BA 44/45/47/6/9) in PD is weaker. We expected that reduced cortical thickness of the left IFG could have a detrimental effect on tasks that combine executive control and verbal competence.

To verify this hypothesis, we tested idiopathic PD patients with asymmetric clinical expression (mild-moderate stage of disease).

On the basis evidence supporting differential patterns of cognitive abilities in clinically asymmetric PD (Verreyt et al., 2011), we hypothesized preferential left hemispheric impairment in verbal tasks, such as word production. In particular, we expected that right PD patients (RPD) (with prevalent left hemisphere nigrostriatal hypofunctionality, henceforth RPD-LH), would be less accurate and slower than left PD patients (LPD) (with prevalent right hemisphere nigrostriatal hypofunctionality, henceforth LPD-RH).

In relation to the executive nature of the word selection tasks, we also expected to detect an association between the volume of specific cortical brain regions of the LH and accuracy and RTs in nouns production (that must be selected among a larger number of alternatives than the verbs). The left IFG in fact, as above mentioned, has been considered a crucial region for selection processing and post lexical control during word production (Badre et al., 2005; Moss et al., 2005; Thompson-Schill and Botvinick, 2006; Siri et al., 2008).

## Materials and methods

### Participants

Twenty-one PD participants (11 males and 10 females) and 19 healthy controls (HC) participated to the study. All subjects were right handed at the Edinburgh Handedness Inventory (Olfield, 1971). Persons with PD were consecutively recruited from the IRCCS Don Carlo Gnocchi Foundation—Neurological Unit (Milan, Italy). To be included in the study, the subject had to meet the following criteria: (1) diagnosis of probable PD according to the United Kingdom Parkinson's Disease Society Brain Bank (Hughes et al., 1992); (2) positive DAT scan; (3) mild to moderate stage of the disease with a scoring between stages 1 and 2.5 of the Modified Hoehn and Yahr (H&Y) Scale (Goetz et al., 2004); (4) at least 8 years of education; (5) Italian native language; (6) no decline in cognitive ability reported by either the patient or informant, or observed by the clinician, (7) Mini-Mental State Examination (MMSE) score greater than or equal to 24; (8) stable drug therapy with L-Dopa (alone or in association with dopamine agonistics, catechol-O-methyltransferase inhibitors, monoamine oxidase inhibitors, and anticholinergic drugs).

Exclusion criteria were: clinical signs satisfying criteria of other neurological disorders, including possible atypical parkinsonisms; secondary or iatrogenic parkinsonism; major psychiatric illnesses excluding presence of mild-moderate depression; claustrophobia.

All PD were characterized using the H&Y scale and Unified Parkinson's Disease Rating Scale (UPDRS)—motor part III (Goetz et al., 2008). To classify left- or right-dominance of symptoms, scores from each item on the UPDRS Part III that contained both a right and a left side component (e.g., finger taps, hand movements, rigidity of extremities, etc) were extracted, which provided left and right motor subscores for each individual. Then, the lateralization score was computed by subtracting the total symptom score from the right side from the total symptom score from the left side and patients were clinically clustered in RPD-LH (*N* = 12) and LPD-RH (*N* = 9). The Levodopa Equivalent Daily Dose (LEDD) (Tomlinson et al., 2010) was also calculated.

A group of 19 HC matched for age, sex, and years of education formed the control group. Demographical data of both groups are detailed in Table 1.

**Table 1:** Demographic and clinical characteristics of HC (healthy controls) and PD (Parkinson's disease) groups of participants.

	**HC**	**PD [*N* = 21]**		**Group comparison**
	**[*N* = 19]**	**LPD-RH [*N* = 9]**	**RPD-LH [*N* = 12]**	**[*p*-value]**
Age [Mean ±*SD*]	65.53 ± 7.83	66.89 ± 9.06	68.33 ± 7.60	0.640 (∧)
Years of education [Mean ±*SD*]	13.68 ± 4.01	13.78 ± 4.18	12.00 ± 3.91	0.472 (∧)
Sex [M/F]	11/8	4/5	7/5	0.819 (#)
Initial symptoms				0.667 (#)
Tremor, *n*		6	5	
Bradykinesia, *n*		1	2	
Rigidity, *n*		1	2	
Gait difficulty, *n*		0	2	
Other, *n*		1	1	
H&Y		1.67±.66	1.63 ± 0.48	0.869 (^*^)
Disease duration		34.67 ± 20.05	51.58 ± 45.85	0.316 (^*^)
**UPDRS III**				
Motor subset score		19.56 ± 11.39	20.58 ± 8.64	0.816 (^*^)
Left-dominance of symptoms		10.22 ± 6.28	4.42 ± 2.50	0.009 (^*^)
Right-dominance of symptoms		2.78 ± 3.42	9.42 ± 2.94	<0.001 (^*^)
Index of lateralization		−7.44 ± 4.39	5.00 ± 1.48	<0.001 (^*^)
**PD MEDICATIONS**				
LEDD		193.00 ± 147.48	311.58 ± 236.51	0.203 (^*^)
MAO-B inih (Sel, Ras), *n*		7 (5,2)	9 (4,5)	0.712 (#)
L-Dopa, *n*		2	8	0.115 (#)
DA (Rop, Pram, Rot), *n*		6 (2,3,1)	4 (–,2,2)	0.488 (#)

RPD-LH, PD with prevalent left hemisphere damage; LPD-RH, PD with prevalent right hemisphere damage; H & Y, Modified Hoehn and Yahr (H&Y) Scale; UPDRS III, Unified Parkinson's Disease Rating Scale—motor part III; LEDD, levodopa equivalent dose; Sel, Selegiline; Ras, Rasagiline; DA, Dopamine-agonist; Rop, Ropinirolo; Pram, Pramipexole; Rot, Rotigotine; (∧)ANOVA; (#)Chi Square statistic; and (

* *)Independent samples t-test*.

The present study was approved by the scientific and Ethics Committee of Don Gnocchi Foundation in accordance with the Helsinki Declaration and all participants gave written informed consent to participate to the study.

### Measures

#### Neuropsychological assessment

PD patients underwent an extensive neuropsychological assessment of general cognitive efficiency (Mini Mental State Examination, MMSE; and Montreal Cognitive Assessment, MoCA), language (object and action oral naming; phonological fluency; semantic fluency); verbal and spatial memory (Immediate and Delayed Recall of 15 words; Free and Cued Selective Reminding Test-FCSRT; Rey-Osterrieth figure recall), verbal and spatial short term memory (verbal span and Corsi's test), intelligence (Raven Colored Matrices), praxis (Rey-Osterrieth figure copy), attention and executive functions (Trail making test, TMT part A, B and B-A; Attentional Matrices; Stroop test; Modified Wisconsin Card Sorting test, MCST), lasting about 2 h, in two sessions. Neuropsychological assessment of HC was limited to MMSE, MOCA, phonological and semantic fluency and TMT.

All neuropsychological tests were corrected for age and education using normative Italian values. The three groups of subjects were matched for age and education. Bonferroni correction for multiple comparisons was applied to set significance.

#### MRI acquisition and data analysis

All 40 subjects received a structural MRI examination. MRI sessions were performed on a 1.5 T scanner (Siemens Magnetom Avanto, Erlangen, Germany) and included [a] 3D T1 MPRAGE scan (TR/TE = 1,900/3.37 ms, FoV = 192 × 256 mm^2^, voxel size 1 mm isotropic, 176 axial slices), to perform cortical and subcortical measurements; [b] conventional anatomical sequences (PD-T2, FLAIR) in order to exclude patients showing macroscopic brain lesions or white matter hyperintensities outside the normal range (Vale et al., 2015). In particular, subjects who presented with one or more macroscopic hyperintensities on T2-weighted scans located in the deep with matter and/or more than five periventricular hyperintensities were excluded.

**Figure 1:**
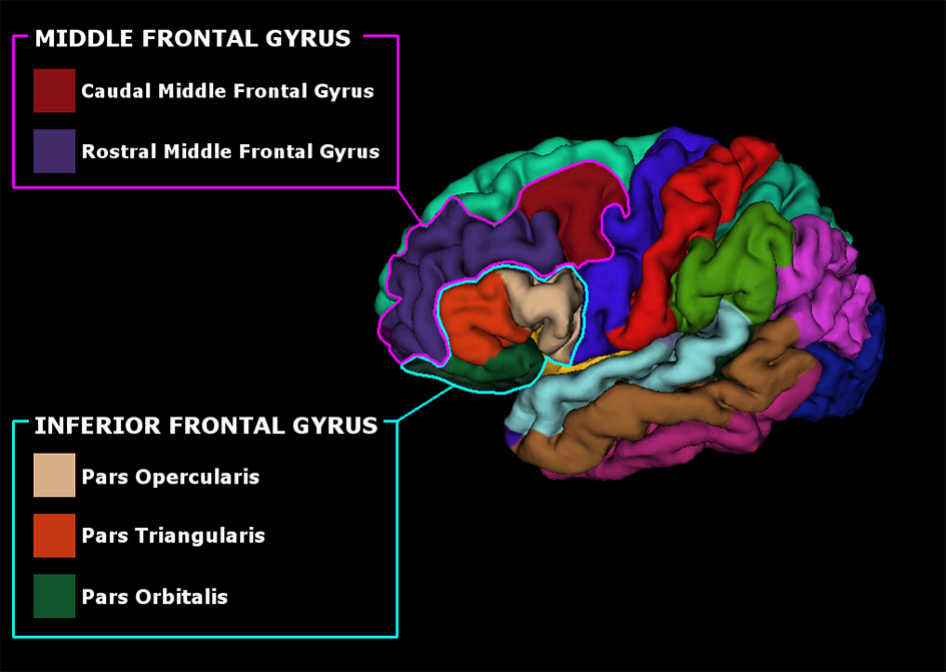
Pial surface reconstruction representing the parcellated subregions of the Inferior Frontal Gyrus (IFG) and Middle Frontal Gyrus (MFG).

Anatomical high-resolution images (3D T1) were segmented and parcellated using Freesufer's recon-all standard pipeline (Dale et al., 1999; Fischl et al., 1999, 2002). Briefly, after brain-extraction, high-resolution 3D T1 images were registered to standard space and the gray/white and gray/cerebrospinal fluid borders were computed (Dale et al., 1999; Fischl et al., 1999, 2002). Quality control checks were performed at all steps of the pipeline and manual corrections were made if necessary. Cortical parcellations were made according to Desikan atlas (Desikan et al., 2006) and thickness measurements were collected from bilateral regions of interest (ROIs). ROIs were selected based on the literature (Alexander et al., 1986; Owen, 2004): the pars opercularis (BA 44), triangularis (BA 45), and orbitalis (BA 47) of the IFG of both the hemispheres and the immediately adjacent regions such as the rostral and caudal middle frontal gyrus (BA 46, BA 9, and 10), see Figure 1. Volumetric measurements were also collected for three PD-related subcortical regions (Lewis et al., 2016): caudate, putamen, and globus pallidus, bilaterally using FreeSurfer subcortical segmentation streamline. Mean Surface Area (MSA) and Total Intracranial Volume (TIV) was also computed for each subject and inserted as covariates in the subsequent statistical analyses to account for differences in brain size; age and sex were included as covariates as well.

### Stimuli

Two lists of words were selected (see Marangolo et al., 2003): 144 verbs (e.g., “osservare” [to observe]), and 144 corresponding derived nouns (e.g., “osservazione” [observation]).

Experimental tasks consisted in the production of morphologically related words: in the derivation task, verbs represented the input stimuli and nouns were the targets (VN: noun from verb); while in the generation tasks, nouns were the inputs and verbs were the targets (NV: verb from noun). In the derivation task, over 90% of the participants produced the same derived noun confirming it as the expected target response; in this condition any other answer was considered as an error. In the generation task, the correspondent verb-base form was the only correct response.

Derived nouns did not differ from verbs in length (nouns: *M* = 8.34, *SD* = 2.56; verbs: *M* = 8.12, *SD* = 1.50), but the two lists of items were different in frequency of use (Istituto di Linguistica Computazionale, CNR, Unpublished Manuscript; see also Marangolo et al., 2003): in fact, the target nouns, that are expected to be associated to a worse performance, were more frequent (*M* = 50.74, out of 1.5 million occurrences; *SD* = 106.05) than the corresponding target verbs (*M* = 28.85; *SD* = 68.99; *t* = 2.08, *p* < 0.04). Note, however, that the difference in frequency went against the hypothesis of worse performance for nouns derived from verbs. Furthermore, the two sets of stimuli differed for the number of alternatives among which the participant had to select his/her response to the stimulus. This variable, which was decisive to test our hypothesis, was estimated taking into account the number of word-types that share the root with the input word and are likely to be involved in processing the response. These word-types are listed in the corpus of written Italian by Bertinetto et al. (2005) (CoLFIS, http://www.istc.cnr.it/en/grouppage/colfis). In the case of verb input (derivation task), there were several alternatives among which the target was to be selected (range: 1–8; *M* = 3.1; *SD* = 1.6), whereas for noun input (generation task) the number of alternatives was 1, as from a noun target only one verb base can be retrieved. Finally, another variable whose influence on the derivation task was considered is the presence of words more frequent than the target in the list of alternatives. For instance, from the verb “abitare” [to reside], the noun produced by all participants (that is, the target) was “abitazione” [residence], but the most frequently derived noun from this verb, listed in the CoLFIS, is “abitante” [resident].

### Procedure

Participants were first screened using a reading test consisting in a set of seven words, different from the stimuli of the experiment, to exclude visual perceptual disorders that could interfere with the cognitive performance.

The input stimuli were presented in two blocks for each task, i.e., two blocks of 72 verbs (144 items for derivation task) and two blocks of 72 nouns (144 items for generation task), in a random order both within and between blocks. Each block was preceded by a training session, where participants were carefully instructed to produce as quickly and accurately as possible the expected target. For example, in the NV generation task participants were instructed to turn the noun into the corresponding infinitive form of the verb. For example, if the noun “sentimento” [feeling] appeared on the display, they were asked to produce the verb “sentire” [to feel] as quickly as possible, considering that the time elapsed from the appearance and the onset of the response was recorded through the microphone. In the VN derivation task, participants were instructed to turn the verb into the corresponding noun. For example, if the verb “partire” [to depart] appeared on the display, they were asked to produce the noun “partenza” [departure] as quickly as possible. During the training session, the examiner could offer a feedback on the accuracy of participant's response. Once the actual task started, the examiner could not give any indication. The administration of the entire experimental session lasted about 60 min with a pause from each block.

Stimuli were presented by SuperLab pro Software (Cedrus, Phoenix, Arizona), on the center of the video display one at time, using a size 60, bolded black font. The experiment was conducted in a quiet room and subjects were seated at a distance of 40 cm from the screen. Each block started with the appearance of the word “via” [start] on the screen and ended with the word “fine” [the end]. The trial sequence started with a blank background for a duration of 250 ms, after that a fixation point appeared for a duration of 750 ms and then the input word for a duration of 5,000 ms. SuperLab pro Software can register reaction times (RTs), which is the latency from the appearance of the word on the computer screen and the onset of response by the participants. These measures were reported in an Excel worksheet generated automatically by SuperLab software. The examiner manually scored response accuracy. Patients treated with L-DOPA were evaluated in “off” state.

### Statistical analyses

#### Behavioral data

Non-parametric analyses were carried out on accuracy and RTs, to assess differences among the three groups of participants (HC, RPD-LH, and LPD-RH) in the two morphology tasks (generation vs. derivation task). To estimate the interaction effect of group by task on accuracy, Chi-squared statistic was carried out on corrected responses. As for RTs, comparisons between the two tasks were assessed through Wilcoxon's test within each group, whereas differences among the three groups were performed through Kruskal–Wallis' test within each task.

Effects of psycholinguistic features on accuracy, computed as the proportion of correct responses after removing technical failures and out-of-time responses, were analyzed through mixed-effects logistic models (Jaeger, 2008). Mixed-effects regression models (Baayen et al., 2008) were carried out to better understand the role of psycholinguistic features of input- and target-words in determining the effects tested in previous analysis on (log-transformed) RTs: along with the variable group, also input frequency, target frequency, target length, number of alternatives, and number of alternatives more frequent than target were tested as fixed effects. In these mixed-effects models, participants and items were tested as random intercepts, along with other three variables that can randomly vary within tasks: (a) phonetic transparency of derivation; (b) presence of homography between the derivative noun and a verbal inflected form; (c) input length in graphemes.

All fixed effects tested on latency were considered in the logistic models on accuracy, but only participants and items were carried out as random intercepts, as computational limits for logistic models do not allow to test the other three random effects (transparency, homography, and length of the input) all together.

#### MRI data

##### MRI between-groups whole-brain comparison

In order to determine if our MRI brain measurements (cortical thickness, subcortical volumes) were modeled by a normal distribution, normality tests (skewness and kurtosis ranges and Q-Q plots) were performed for each variable using Statistical Package for Social Sciences (SPSS, IBM Corporation), version 24. Left and right sides of the same area have been considered separately.

In order to verify the presence of brain atrophy, cortical brain thickness was compared between HC (*N* = 19) and PD groups (*N* = 20, a subject was excluded due to motion artifacts) at a whole-brain level using QDEC, (https://surfer.nmr.mgh.harvard.edu/fswiki/Qdec), running on Freesurfer. A two-sample *t-*test was performed, inserting age and gender as covariates, results were considered as statistically significant when surviving the *p* < 0.05, corrected for multiple comparison (FDR). Then, a direct comparison was performed between RPD-LH (*N* = 11) and LPD-RH (*N* = 9) subgroups in order to assess anatomical brain differences between the two subgroups. Also in this case, age and gender were inserted as nuisance covariates and results were considered as statistically significant with a *p* < 0.05_FDR−corrected_. Moreover, direct comparisons were performed for subcortical volumes (for each brain side) using ANCOVA: group of participants as between factor (3 levels: HC, RPD-LH, LPD-RH) and TIV, age and sex as covariates.

##### MRI correlation analysis

Partial correlations were computed between NV [both accuracy and log-transformed RTs] and cortical thickness of the considered ROIs and between VN [both accuracy and log-transformed RTs] and cortical thickness. Mean surface areas, age and sex were inserted as covariates, to account for brain size differences. Partial correlations were also computed between NV [both accuracy and log-transformed RTs] and subcortical volumes. Correlations were computed separately in [a] HC group; [b] RPD-LH; [c] LPD-RH.

Results were considered as statistically significant at *p* < 0.05 after Benjamini–Hochberg procedure for controlling the false discovery rate (FDR) in multiple testing (Benjamini and Hochberg, 1995).

## Results

### Demographical data and neuropsychological tests

Participants were matched on sex, age, and years of education. Regarding the PD subgroups, there was no statistically significant difference in symptoms at onset, disease duration, H&Y score, and UPDRS motor subset score between LPD-RH and RPD-LH patients, which ensures that disease severity was uniform between subgroups (see Table 1). Moreover, no significant differences in levodopa equivalent daily dose (LEDD) and kind of PD medication between the two PD subgroups were found (see Table 1 for details).

Results showed that the LPD-RH and RPD-LH groups had no significantly lower adjusted scores than the HC group on any neuropsychological tests, except for the TMT sub-test part A, where LPD-RH were slower than HC [*F*_(2, 37)_ = 7.585, *p* = 0.002]. No other difference reached statistical significance between HC and PD groups and between PD groups (see Table 2).

**Table 2:** Adjusted and row scores (when adjusted scores are not available) obtained by HC (healthy controls) and PD (Parkinson's disease) groups on neuropsychological tests.

**Neuropsychological test [Mean ± Standard Deviation]**	**HC [*N* = 19]**	**PD [*N* = 21]**		**Group comparison [*p*-value]**
		**LPD-RH [*N* = 9]**	**RPD-LH [*N* = 12]**	
MMSE (0–30)	27.79 ± 1.34	27.56 ± 1.88	27.24 ± 1.68	0.636 (∧)
MoCA (0–30)	26.16 ± 2.64	23.35 ± 3.29	24.22 ± 3.65	0.077 (∧)
Phonological fluency	37.14 ± 6.44	36.96 ± 10.71	38.19 ± 8.41	0.923 (∧)
Semantic fluency	45.89 ± 5.01	44.67 ± 12.13	43.42 ± 7.53	0.690 (∧)
TMT part A	26.84 ± 15.85	58.78 ± 28.80	42.67 ± 20.60	**0.002** (∧)
TMT part B	58.79 ± 31.70	116.11 ± 79.20	94.33 ± 113.04	0.150 (∧)
TMT part B-A	31.89 ± 26.56	57.56 ± 56.30	58.00 ± 98.09	0.431(∧)
Attentional matrices (0–60)		49.39 ± 8.12	49.58 ± 4.92	0.955 (#)
Verbal span forward (0–9)		6.21 ± 0.77	6.01 ± 1.22	0.697 (#)
Verbal span backward (0–9)		4.45 ± 0.70	4.99 ± 1.20	0.305 (#)
Corsi's test forward (0–9)		5.33 ± 0.99	5.29 ± 0.84	0.929 (#)
Corsi's test backward (0–9)		4.70 ± 1.19	4.52 ± 0.97	0.732 (#)
Immediate recall of 15 words (0–75)		48.99 ± 5.38	49.70 ± 9.40	0.859 (#)
Delayed recall of 15 words (0–15)		10.97 ± 2.66	10.46 ± 3.47	0.748 (#)
Rey-Osterrieth figure copy (0–36)		29.84 ± 6.16	29.65 ± 4.92	0.942 (#)
Rey-Osterrieth figure recall (0–36)		15.94 ± 8.85	15.38 ± 6.52	0.878 (#)
FCSRT IFR (0–36)		28.61 ± 3.78	29.05 ± 3.96	0.807 (#)
FCSRT ITR ^*^ (0–36)		35.33 ± 1.00	35.50 ± 0.97	0.717 (#)
FCSRT DFR (0–12)		10.43 ± 1.26	10.69 ± 1.45	0.687 (#)
FCSRT DTR ^*^ (0–12)		11.89 ± 0.33	11.90 ± 0.32	0.941 (#)
FCSRT ISC (0–1)		0.84 ± 0.33	0.88 ± 0.31	0.767 (#)
Raven Colored Matrices (0–36)		30.94 ± 5.32	30.50 ± 4.66	0.848 (#)
MCST ^*^ (number of completed categories)		5.57 ± 1.62	6.00 ± 1.41	0.570 (#)
MCST (number of perseverative errors)		3.96 ± 5.44	2.40 ± 3.46	0.478 (#)
Stroop test—time interference effect		18.62 ± 15.64	15.61 ± 10.22	0.636 (#)
Stroop test—error interference effect		0.00 ± 0.00	0.83 ± 2.52	0.405 (#)
Object oral naming (0–30)		28.43 ± 1.51	28.11 ± 1.17	0.642 (#)
Action oral naming (0–28)		26.86 ± 1.68	25.67 ± 2.00	0.227 (#)

LPD-RH, (PD with prevalent right hemisphere nigrostriatal hypofunctionality); RPD-LH, (PD with prevalent left hemisphere nigrostriatal hypofunctionality); MMSE, Mini Mental State Examination; MoCA, Montreal Cognitive Assessment; TMT, Trail Making Test; FCSRT, Free and Cued Selective Reminding Test; IFR, Immediate Free Recall; ITR, Immediate Total Recall; DFR, Delayed Free Recall; DTR, Delayed Total Recall; CSI, Cueing Sensitivity Index; MCST, Modified Wisconsin Card Sorting test; (∧) ANOVA statistic; (#) Independent samples t-test;

* *Row scores. Significant p-value (p < 0.05) are highlighted in bold font*.

### Behavioral tasks

**Figure 2:**
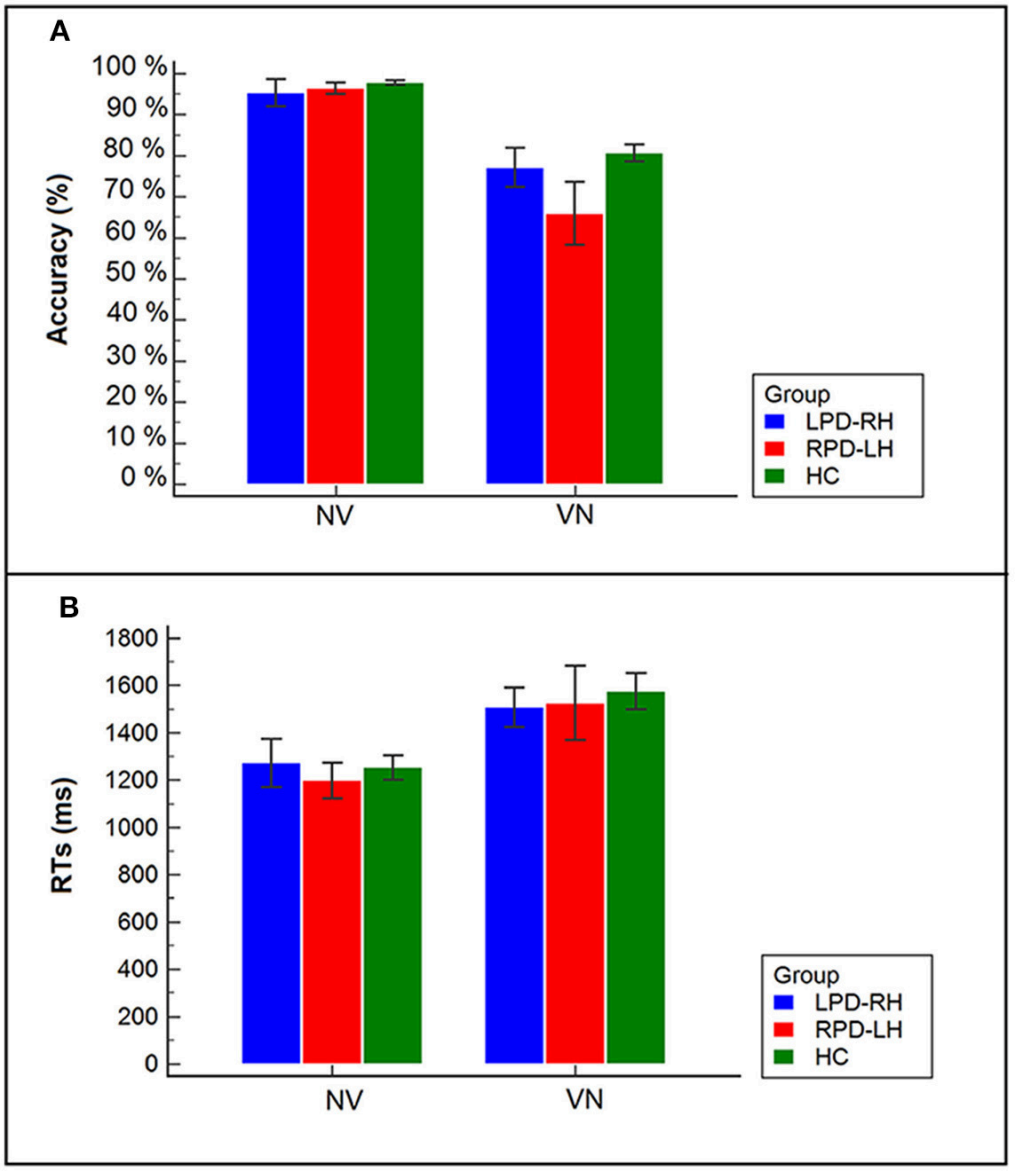
**(A)** Accuracy of PD patients and controls in the two tasks (VN, noun from verb; NV, verb from noun); **(B)** Reaction times (RTs) of PD patients (LPD-RH; RPD-LH) and controls (HC) in the two tasks (NV, verb from noun; VN, noun from verb).

Technical failures and responses given after the time limit of 5,000 ms (LPD-RH patients: 13%; RPD-LH: 13.8%; HC: 6.6%) were excluded from the analyses. Accuracy and mean RTs are shown in Figure 2 and Table 3.

**Table 3:** Mean (M) and Standard Deviation (SD) RTs (ms) and accuracy in generation (NV) and derivation (VN) tasks by groups.

**Group**		**Task**			
		**NV**		**VN**	
		***M***	***SD***	***M***	***SD***
LPD-RH	RTs	1,273	305.5	1,508	251.7
(*N* = 9)	Accuracy	95%		77%	
RPD-LH	RTs	1,197	263.9	1,526	540.3
(*N* = 12)	Accuracy	96%		66%	
HC	RTs	1,252	226.9	1,576	340.3
(*N* = 19)	Accuracy	98%		80%	

#### Accuracy

The interaction between group and task proved to be significant (χ^2^ = 21.287, df = 2, *p* < 0.001): all participants showed a lower percentage of accuracy in the derivation (74.5%) (e.g., osservare [to observe] –> osservazione [observation]) than in the generation task (96.5%). However, it is worth noting that RPD-LH patients had a level of accuracy in derivation task (65.9%; standardized residual = −2.9) lower not only than controls (80.6%), but also than LPD-RH patients (77.1%), who did not significantly differ from the HC group.

The number of alternatives and the number of alternatives more frequent than the target proved to exert significant effects (*b* = −0.4974, *z* = −7.380, *p* < 0.001; *b* = −1.0249, *z* = −6.033, *p* < 0.001, respectively): the higher is their value, the lower is the percentage of accuracy showed by all participants. The inhibitory effect of input frequency was significant, as well (*b* = −0.1414, *z* = −2.08, *p* = 0.037). On the contrary, target frequency had a facilitatory effect: the higher the frequency, the higher the percentage of accuracy (*b* = 0.3526, *z* = 5.246, *p* < 0.001).

#### Latency

Analysis on RTs was carried out only for trials in which the word was correctly produced (accuracy for LPD-RH: 86.1%; for RPD-LH: 81.1%; for HC: 89.3%).

Wilcoxon's test showed that the derivation task (VN), which involves selection among several alternatives, was more difficult than the generation task (NV), which can be done by only retrieving the correct word-base, in each group (HC: *z* = −3.823, *p* < 0.001; LPD-RH: *z* = −2.429, *p* = 0.015; RPD-LH: *z* = −3.059, *p* = 0.002): all participants were much slower (*M* = 1,536 ms, *SE* = 65.6) when having to derive a noun from a verb (VN; e.g., *osservare* [to observe] –> *osservazione* [observation]) than when they had to generate the verb base from a derived noun (NV; e.g., *fallimento* [failure] –> *fallire* [to fail]) (*M* = 1,240 ms, *SE* = 45.5). No significant difference was observed among groups within each task (Kruskal–Wallis' test for NV: χ^2^ = 0.725, *df* = 2, n.s.; Kruskal–Wallis' test for VN: χ^2^ = 1.577, *df* = 2, n.s.).

**Figure 3:**
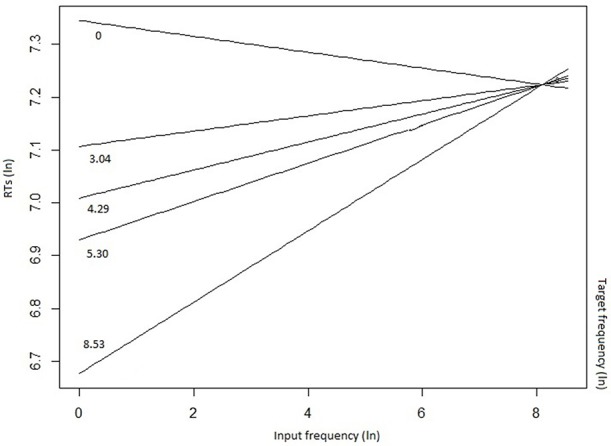
Input frequency by target frequency interaction on reaction times (RTs).

The mixed-effects model that best fitted the whole data set (Table 4) confirmed the inhibitory effect of number of alternatives (*b* = 0.019, *t* = 2.934, *p* = 0.0036), and of the number of alternatives more frequent than the target (*b* = 0.076, *t* = 3.861, *p* < 0.001). These results show that, in the case of a high competition among several lexical entries, in particular in presence of high-frequency competitors (including the input word itself), the target selection is slowed down. The effect of the presence of high-frequency competitors was moderated by the group (LPD-RH^*^number of alternatives more frequent than the target: *b* = 0.079, *t* = 4.491, *p* < 0.001), as, in the *post-hoc* analyses, the LPD-RH's performance seemed to be minimally affected by this feature (*b* = −0.009, *t* = −0.390, *p* = 0.69), whereas both HC (*b* = 0.06, *t* = 3.09, *p* = 0.002) and RPD-LH (*b* = 0.08, *t* = 3.203, *p* = 0.001) show a reliable inhibitory effect. Finally, results show that the higher the target frequency, the faster was the participant's response (*b* = −0.078, *t* = −5.851, *p* = < 0.001), but this effect influenced the performance in interaction with input frequency (*b* = 0.009, *t* = 3.371, *p* < 0.001). Figure 3 shows that target frequency had a stronger facilitatory effect when input frequency was low (left side of the figure), whereas as input frequency increased, the inhibition on the target production was stronger, irrespective to the target frequency.

**Table 4:** Mixed-effects model on (log-transformed) reaction times (RTs): random effects, estimates and *t*-values of fixed effects.

	**Variance**	**Std. Dev**.		
**RANDOM EFFECTS**				
Input	0.01774	0.133190		
Subject	0.04178	0.204396		
Input length	0.00054	0.023370		
Transparency	0.00008	0.008971		
Homography	0.00454	0.067397		
Residual	0.07851	0.280202		
	**Estimate**	**Std. Error**	***t*****-value**	**Pr(>|t|)**
**FIXED EFFECTS**				
(Intercept)	7.326	0.08408	87.131	<0.001
LPD-RH	0.0077	0.08309	0.093	0.926130
RPD-LH	0.0645	0.07573	−0.853	0.399376
Number of alternatives	0.0192	0.00654	2.934	0.003633
Number of alternatives more frequent than target	0.0758	0.01965	3.861	0.000131
Target frequency	−0.07836	0.01339	−5.851	<0.001
Input frequency	−0.01503	0.0281	−1.135	0.257416
LPD-RH^*^N. of alternatives more frequent than target	−0.07942	0.01768	−4.491	<0.001
RPD-LH^*^N. of alternatives more frequent than target	0.01762	0.00882	−0.740	0.459332
Target freq.^*^Input freq.	0.009682	0.002872	3.371	0.000856

The other psycholinguistic variables considered as random variables, i.e., phonetic transparency of the derivation, homography between noun and verb forms, and input length, had negligible effects on latency.

### Brain measurements and correlation analysis

#### MRI between-groups whole-brain comparison

We found no signs of cortical atrophy in the PD group, compared with HC, at a whole-brain level (*p* FDR corr < 0.05). The direct comparison between RPD-LH and LPD-RH subgroups also revealed no differences in the cortical thickness associated with laterality of the pathology (*p* FDR corr < 0.05).

No statistically significant differences were also found in subcortical volumes (caudate, putamen, and globus pallidus, bilaterally) when including TIV, age, and sex as covariates (ANCOVA).

#### Correlation analysis

##### Accuracy

**Figure 4:**
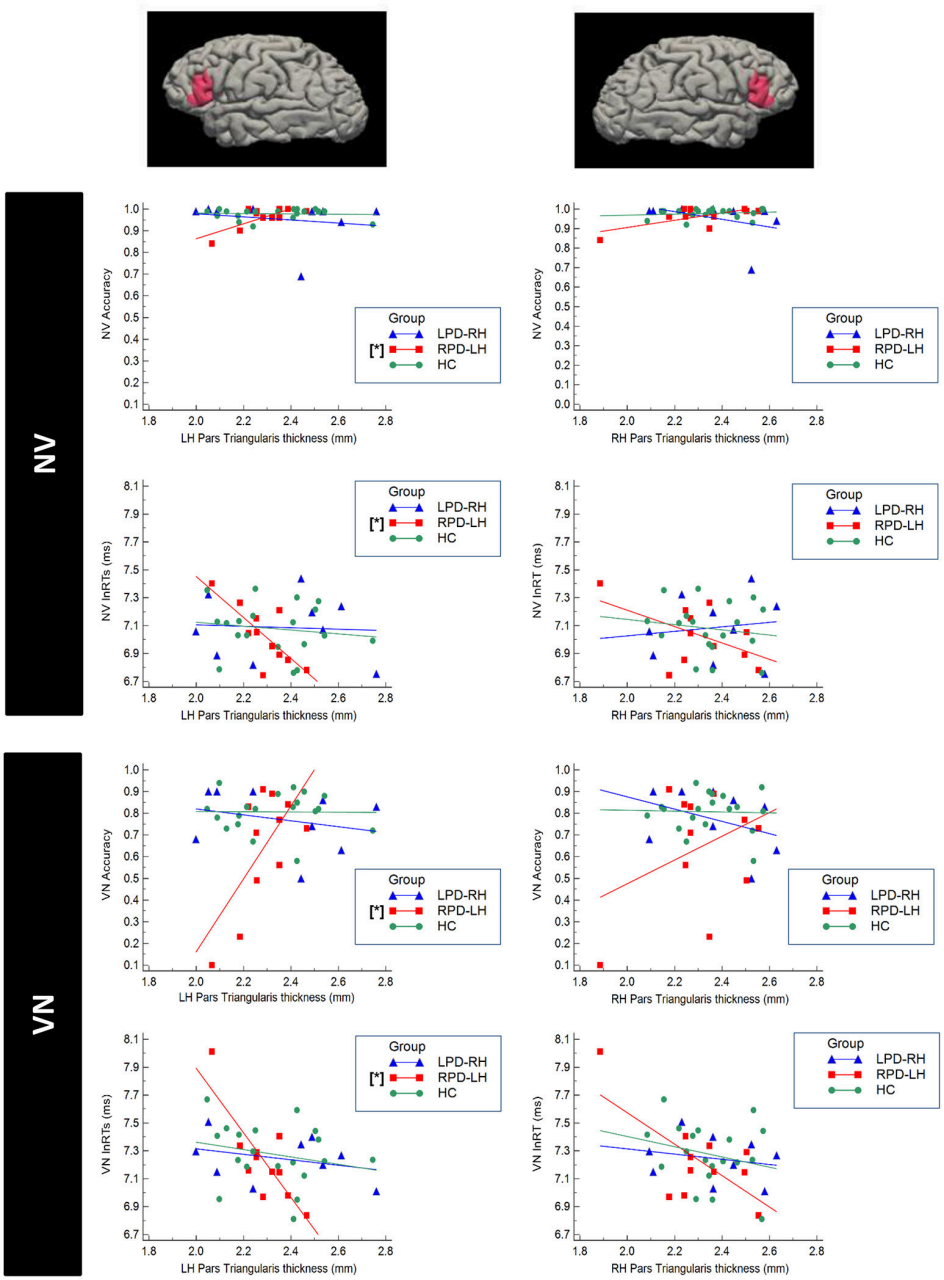
Scatterplot representing the correlation between cortical thickness of the Pars Triangularis of the Inferior Frontal Gyrus (IFG) and performance at the verbal test in HC (healthy controls), RPD-LH (PD with prevalent left hemisphere nigrostriatal hypofunctionality), LPD-RH (with prevalent right hemisphere nigrostriatal hypofunctionality. Significant correlations (*p* < 0.05 FDR-corrected) are marked by [^*^]. NV, verb from noun task; VN, noun from verb task; lnRTs, (log-transformed) reaction times.

Significant correlations were found only between accuracy and respectively RTs and cortical thickness in PD group with left hemisphere damage (Figure 4). In more details, RPD-LH showed significant partial correlations between the NV and VN accuracy respectively and left pars triangularis (NV: *r* = 0.717, *p* = 0.045; VN: *r* = 0.873, *p* = 0.010).

No other correlation did emerge in LPD-RH and HC.

##### RTs

Significant inverse partial correlation also emerged between NV and VN RTs and left pars triangularis in RPD-LH (NV: *r* = −0.845; *p* = 0.011; VN: *r* = −0.872; *p* = 0.010; Figure 4). No other significant correlation did emerge in any group.

Finally, no correlation was found in any group between noun and verb production and volume of subcortical regions (caudate, putamen and globus pallidus, bilaterally). For a detailed report of all correlations see Supplementary Materials.

## Discussion

In the present study we aimed to replicate previous results (Silveri et al., 2018) in a new cohort of well-selected PD patients with a clear symptom side predominance (left or right), at a mild to moderate stage of the disease, positive DAT scan and with no decline in cognitive functioning. Indeed, our results confirm that noun and verb production may be differentially impaired when evaluated by a morphological paradigm that allows for keeping under control the number of alternatives among which operate word selection.

More specifically, in VN derivation tasks, which involve selection among many alternatives, we found two main results. First of all, the VN accuracy was lower than NV accuracy in all groups and RPD-LH patients were significantly less accurate than LPD-RH, who did not differ from HC. Furthermore, the greater the number of alternatives and number of alternatives more frequent than the target, the lower was the accuracy in all subjects; while the higher the frequency of the target, the higher was the accuracy. Secondly, the responses in VN derivation tasks were slower than in NV generation tasks, with no difference among the three groups of subjects. The presence of high-frequency competitors in derivation tasks did not produce any affect in LPD-RH, while both RPD-LH and HC showed increased latencies of response, and the frequency of the target, in interaction with the input frequency, reduced the latency of response in all subjects.

These results are consistent with the hypothesis that word production requires resolving competition among alternatives (Usher and McClelland, 2001); when attentional resources decay, as in some neurological conditions such as PD, word production is penalized in relation to the number of alternatives among which the selection is made. It is worth noting that the decay of accuracy is principally on the account of the PD population, but only when the left hemisphere (RPD-LH) is involved, as expected on the base of the linguistic nature of the task. However, the nature of the task by itself does not seem sufficient for the emergence of a word production deficit. In fact, when it is the verb instead of noun to be produced, both PD groups and HC perform similarly. This result suggests that the production deficit emerges as a function of the difficulty of the task (attentional demand for the selection from many alternatives) in the presence of a left hemisphere dysfunction. Briefly said, nature of the task and attentional components converge in producing the results (reducing accuracy in noun production) only when the left hemisphere is damaged. In the mild to moderate phase of PD, executive dysfunction is one of the principal non-motor symptoms; the executive function controls the cognitive behaviors and its effect becomes apparent only when subjects are involved in specific cognitive tasks. The dysexecutive syndrome, therefore, in our case, acquires the expression of a language deficit.

Presence of high-frequency competitors to the target resulted in a detrimental effect on latency but only in HC and RPD-LH. Thus, in respect to previous reports (Crescentini et al., 2008; Silveri et al., 2018) controlled retrieval was to some extent impaired in PD, but differentially in LPD-RH and RPD-LH; the former group seemed insensitive to the interference. Taken together, these results might suggest that left and right hemisphere are different when bottom up inhibition of irrelevant information is requested. Such an “insensitivity” of the LPD-RH to the presence of interference is difficult to explain at this time and requires further confirmation in a larger sample of subjects. However, it is consistent with experimental data indicating a different contribution of the inferior regions of the left and right prefrontal cortex in response inhibition (Baglio et al., 2011; Dambacher et al., 2014) and impulse control (Boes et al., 2008). Impulsive behavior is a relatively frequent symptom in PD and is mostly in relation to dopamine treatment (Weintraub and Claassen, 2017) but the effects of the hemispheric side on the impulse control have not been explicitly explored in this pathology and further studies should address this issue in the future.

For what concerns the neuroimaging, we investigated if accuracy and RTs obtained in behavioral tasks were correlated with the morphometric status of the specific brain regions thought to support word selection processes, such as the left IFG. No difference was found between the three groups in the cortical thickness for all the selected ROIs. However, significant correlations between the cortical thickness and word production of both noun and verb emerged in patients with RPD-LH in the IFG of the left hemisphere. No correlation instead, was found between word production and thickness of the rostral and caudal middle frontal regions and with the volume of the subcortical regions considered. Lack of correlation in this case could be explained by considering that these regions might be implicated in higher level control functions (Owen, 2004) less influenced by the linguistic components compared to the IFG.

Both noun and verb production generated significant correlations with a specific subregion of the left IFG, in particular the pars triangularis, although a tendency toward significance was observed also with the pars orbitalis. We would expect, however a greater influence of the executive disorder and that noun, more than verb production, would correlate with the thickness of these regions. Thus, the critical factor for significant correlations to emerge, seems to be the language deficit rather than the dysexecutive disorder.

The element that should not be underestimated to account for these results is that the subregions of the IFG whose thickness correlated with word production belong to the so called Broca's complex (Hagoort, 2005), including BA 44, 45, and 47. BA 44 is the core of the Broca's complex, while more rostral areas such as BA 45 and 47 assume a more specific role in the executive control (Ardila et al., 2016a). Thus, IFG-Broca's complex is not only involved in selection among competing alternatives but underlies the complex of functions related to language production (see Ardila et al., 2016b for discussion). Word production “*per se*,” and thus also for verb (that in our experimental condition does not require selection processes), is subtended by the IFG. Based on our analyses, the association between accuracy and RTs and cortical thickness seems to some extent more evident in the rostral regions (BA 45, BA 47) than in BA 44, regions which are more involved in executive control (Ardila et al., 2016a), However, our data do not allow to disentangle whether the association is a result of demand on executive resources, or whether associations emerge because the IFG is also part of the language production system.

Interestingly, the emergence of significant correlations in RPD-LH between IFG thickness and the production of the verb in an experimental condition that makes verb production easier than nouns leaves open another possible interpretation. It might be that the word class “verb” (at least some classes of verbs) are represented in neural substrates deputed to movement and movement control (Fernandino et al., 2013) such as the corticostriatal network. Our data do not exclude that verb meaning might be “embodied” in cerebral regions related to action representation by the integration of sensorimotor features (Barsalou, 1999). Moreover, other reports (Bocanegra et al., 2015) downsize the role of the executive disorder in producing verb deficit in PD and propose an interpretation that involves the decline of semantic memory or other components of language.

In conclusion, our data confirm that the verb production deficit in PD, often reported in the literature (Péran et al., 2003; Signorini and Volpato, 2006; Colman et al., 2009; Rodríguez-Ferreiro et al., 2009; Silveri et al., 2012; Bocanegra et al., 2015) may be traced back to the dysexecutive syndrome, but other hypotheses cannot be excluded. The linguistic nature of the tasks we adopted in the present study proved to be an influential factor. Decreased task performance was in facts primarily found in PD patients with left hemisphere damage. In agreement with previous reports (Verreyt et al., 2011) our data also indicate that side of the clinical symptomatology should not be underestimated when assessing cognitive functions in PD patients, since it may be a strong predictor of the characteristics of cognitive decline in this pathology.

Finally, we acknowledge that this is an exploratory study considering the relatively small number of participants and that additional investigations are needed to confirm the data.

## Author contributions

SD, FB, DT, and MS: study concept and design; FB and MS: study supervision; RN: subjects' recruiting; SD and FB: acquisition of data; SD, FB, MC, DT, and MS: analysis and interpretation of data; SD, FB, MC, RN, DT, and MS: drafting/revising the manuscript. All the authors approved the final version of the work to be published and agreed to be accountable for all aspects of the work.

### Conflict of interest statement

The authors declare that the research was conducted in the absence of any commercial or financial relationships that could be construed as a potential conflict of interest.
