# Supplementary material for: Selection Processing in Noun and Verb Production in Left- and Right-Sided Parkinson's Disease Patients
Source: Front Psychol. 2018 Jul 20;9:1241. doi: 10.3389/fpsyg.2018.01241 (PMC6062671; doi:10.3389/fpsyg.2018.01241)
Supplement: Supplementary file 1 [file Table_1.docx]

**Table 1:** ANCOVA on subcortical volumes with groups as between factor (HC (healthy controls), RPD-LH (PD with prevalent left hemisphere nigrostriatal hypofunctionality), LPD-RH (with prevalent right hemisphere nigrostriatal hypofunctionality), Total Intracranial Volume (TIV), age and sex as covariates. Statistical significance was set at p<0.05. Abbreviations: L=left hemisphere, R= right hemisphere, df=degrees of freedom.

| **Subcortical volumes** | **Side** | **Statistical test (F)** | **df** | **Group comparison [P value]** | **HC** | | **PD [N=20]** | | | |
| --- | --- | --- | --- | --- | --- | --- | --- | --- | --- | --- |
|  |  |  |  |  |  |  | **LPD-RH** | | **RPD-LH** | |
|  |  |  |  |  |  |  |  |  |  |  |
|  |  |  |  |  | **Mean** | **SD** | **Mean** | **SD** | **Mean** | **SD** |
|  |  |  |  |  | **[N=19]** | | **[N=9]** | | **[N=11]** | |
| Caudate [mm^3^] | L | 0.08 | 2,33 | 0.921 | 3220.35 | 470.64 | 3258.36 | 540.90 | 3227.81 | 422.86 |
|  | R | 0.14 | 2,33 | 0.866 | 3244.87 | 471.36 | 3301.27 | 562.92 | 3245.90 | 451.22 |
| Putamen [mm^3^] | L | 3.01 | 2,33 | 0.063 | 4664.36 | 570.89 | 4157.88 | 1000.21 | 4249.51 | 579.92 |
|  | R | 1.63 | 2,33 | 0.211 | 4295.74 | 528.65 | 3936.56 | 845.65 | 4132.47 | 626.56 |
| Pallidum [mm^3^] | L | 0.01 | 2,33 | 0.985 | 1283.35 | 179.38 | 1328.42 | 331.21 | 1336.15 | 303.71 |
|  | R | 0.19 | 2,33 | 0.829 | 1304.36 | 164.32 | 1303.21 | 219.24 | 1361.75 | 218.12 |
