# Supplementary material for: Selection Processing in Noun and Verb Production in Left- and Right-Sided Parkinson's Disease Patients
Source: Front Psychol. 2018 Jul 20;9:1241. doi: 10.3389/fpsyg.2018.01241 (PMC6062671; doi:10.3389/fpsyg.2018.01241)
Supplement: Supplementary file 2 [file Table_2.docx]

**Table 2:** Partial correlations between cortical thickness and performance at the verbal test in HC (healthy controls), RPD-LH (PD with prevalent left hemisphere nigrostriatal hypofunctionality), LPD-RH (with prevalent right hemisphere nigrostriatal hypofunctionality). Mean Surface Area (MSA), age and sex have been inserted as variables of no interest. Significant correlations (p<0.05 FDR-corrected) are highlighted in bold font. Abbreviations: R=Pearsons' correlation coefficient; NV=verb from noun task; VN=noun from verb task; RTs=reaction times; IFG oper=Inferior Frontal Gyrus pars opercularis; IFG trian=Inferior Frontal Gyrus pars triangularis; IFG orb=Inferior Frontal Gyrus pars orbitalis; MFG rostral=Middle Frontal Gyrus rostral part; MFG caudal=Middle Frontal Gyrus caudal part.

|  |  |  | **LEFT HEMISPHERE** | | | | | **RIGHT HEMISPHERE** | | | | |
| --- | --- | --- | --- | --- | --- | --- | --- | --- | --- | --- | --- | --- |
|  |  |  | **IFG oper** | **IFG trian** | **IFG orb** | **MFG rostral** | **MFG caudal** | **IFG oper** | **IFG trian** | **IFG orb** | **MFG rostral** | **MFG caudal** |
|  |  |  |  | 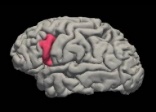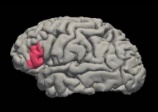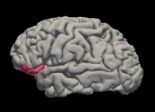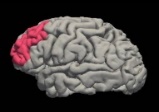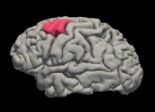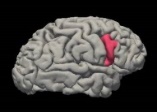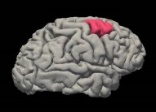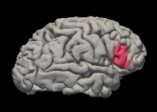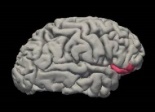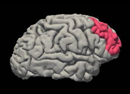 |  |  |  |  |  |  |  |  |
| **RPD-LH** | **NV accuracy** | R | 0.155 | **0.717** | 0.529 | 0.139 | 0.223 | 0.281 | 0.499 | -0.472 | 0.251 | 0.139 |
|  |  | p value | 0.774 | **0.045** | 0.178 | 0.742 | 0.814 | 0.951 | 0.405 | 0.952 | 0.549 | 0.742 |
|  | **VN accuracy** | R | 0.289 | **0.873** | 0.704 | 0.461 | 0.100 | 0.026 | 0.251 | -0.151 | 0.430 | 0.461 |
|  |  | p value | 0.774 | **0.010** | 0.087 | 0.742 | 0.814 | 0.951 | 0.549 | 0.988 | 0.384 | 0.742 |
|  | **NV RTs** | R | -0.122 | **-0.845** | -0.677 | -0.354 | -0.145 | 0.120 | -0.417 | 0.092 | -0.459 | -0.354 |
|  |  | p value | 0.774 | **0.011** | 0.087 | 0.742 | 0.814 | 0.951 | 0.405 | 0.988 | 0.384 | 0.742 |
|  | **VN RTs** | R | -0.217 | **-0.872** | -0.732 | -0.246 | -0.264 | -0.287 | -0.594 | -0.007 | -0.497 | -0.246 |
|  |  | p value | 0.774 | **0.010** | 0.087 | 0.742 | 0.814 | 0.951 | 0.405 | 0.988 | 0.384 | 0.742 |
| **LPD-RH** | **NV accuracy** | R | -0.013 | -0.102 | -0.397 | -0.310 | 0.300 | -0.053 | -0.349 | -0.175 | -0.049 | -0.310 |
|  |  | p value | 0.981 | 0.985 | 0.979 | 0.751 | 0.564 | 0.921 | 0.921 | 0.943 | 0.927 | 0.751 |
|  | **VN accuracy** | R | -0.038 | -0.010 | -0.167 | -0.301 | 0.398 | -0.076 | -0.177 | -0.038 | -0.089 | -0.301 |
|  |  | p value | 0.981 | 0.985 | 0.979 | 0.751 | 0.564 | 0.921 | 0.921 | 0.943 | 0.927 | 0.751 |
|  | **NV RTs** | R | -0.413 | -0.329 | 0.014 | -0.111 | -0.694 | -0.377 | -0.053 | -0.149 | -0.376 | -0.111 |
|  |  | p value | 0.832 | 0.985 | 0.979 | 0.834 | 0.252 | 0.921 | 0.921 | 0.943 | 0.924 | 0.834 |
|  | **VN RTs** | R | -0.701 | -0.576 | -0.274 | -0.501 | -0.880 | -0.626 | -0.435 | -0.211 | -0.634 | -0.501 |
|  |  | p value | 0.484 | 0.924 | 0.979 | 0.751 | 0.084 | 0.736 | 0.921 | 0.943 | 0.704 | 0.751 |
| **HC** | **NV accuracy** | R | 0.347 | -0.114 | -0.030 | -0.205 | 0.076 | 0.005 | 0.020 | 0.110 | -0.170 | -0.205 |
|  |  | p value | 0.404 | 0.811 | 0.912 | 0.611 | 0.780 | 0.987 | 0.944 | 0.686 | 0.761 | 0.611 |
|  | **VN accuracy** | R | 0.145 | -0.195 | -0.200 | -0.214 | -0.286 | -0.070 | -0.332 | -0.483 | -0.401 | -0.214 |
|  |  | p value | 0.592 | 0.811 | 0.912 | 0.611 | 0.740 | 0.987 | 0.840 | 0.232 | 0.496 | 0.611 |
|  | **NV RTs** | R | -0.270 | -0.065 | 0.090 | -0.078 | -0.159 | 0.007 | -0.019 | 0.117 | 0.153 | -0.078 |
|  |  | p value | 0.415 | 0.811 | 0.912 | 0.775 | 0.740 | 0.987 | 0.944 | 0.686 | 0.761 | 0.775 |
|  | **VN RTs** | R | -0.337 | -0.207 | 0.065 | -0.200 | -0.181 | -0.134 | -0.120 | 0.187 | 0.016 | -0.200 |
|  |  | p value | 0.404 | 0.811 | 0.912 | 0.611 | 0.740 | 0.987 | 0.944 | 0.686 | 0.953 | 0.611 |
