# Supplementary material for: Selection Processing in Noun and Verb Production in Left- and Right-Sided Parkinson's Disease Patients
Source: Front Psychol. 2018 Jul 20;9:1241. doi: 10.3389/fpsyg.2018.01241 (PMC6062671; doi:10.3389/fpsyg.2018.01241)
Supplement: Supplementary file 3 [file Table_3.docx]

**Table 3:** Partial correlations between subcortical volumes and performance at the verbal test in HC (healthy controls), RPD-LH (PD with prevalent left hemisphere nigrostriatal hypofunctionality), LPD-RH (with prevalent right hemisphere nigrostriatal hypofunctionality). Total Intracranial Volume (TIV), age and sex have been inserted as variables of no interest. Significant correlations (p<0.05 FDR-corrected) are highlighted in bold font. Abbreviations: R=Pearsons' correlation coefficient; NV=verb from noun task; VN=noun from verb task; RTs=reaction times.

|  |  |  |  |  |  |  |  |  |
| --- | --- | --- | --- | --- | --- | --- | --- | --- |
|  |  |  | Left Caudate | Left Putamen | Left Pallidum | Right Caudate | Right Putamen | Right Pallidum |
| **RPD-LH** | **NV accuracy** | R | 0.305 | 0.265 | 0.576 | 0.234 | 0.228 | 0.360 |
|  |  | p value | 0.425 | 0.569 | 0.379 | 0.544 | 0.555 | 0.519 |
|  | **VN accuracy** | R | 0.382 | 0.255 | 0.451 | 0.361 | 0.250 | 0.432 |
|  |  | p value | 0.413 | 0.569 | 0.379 | 0.544 | 0.555 | 0.519 |
|  | **NV RTs** | R | -0.383 | -0.220 | -0.118 | -0.315 | -0.251 | -0.081 |
|  |  | p value | 0.413 | 0.569 | 0.763 | 0.544 | 0.555 | 0.835 |
|  | **VN RTs** | R | -0.429 | -0.412 | -0.402 | -0.246 | -0.376 | -0.328 |
|  |  | p value | 0.413 | 0.569 | 0.379 | 0.544 | 0.555 | 0.519 |
| **LPD-RH** | **NV accuracy** | R | -0.121 | 0.119 | 0.278 | -0.053 | 0.155 | 0.350 |
|  |  | p value | 0.820 | 0.822 | 0.646 | 0.920 | 0.770 | 0.496 |
|  | **VN accuracy** | R | 0.255 | 0.332 | 0.451 | 0.422 | 0.417 | 0.389 |
|  |  | p value | 0.820 | 0.753 | 0.646 | 0.733 | 0.770 | 0.496 |
|  | **NV RTs** | R | -0.253 | -0.299 | -0.359 | -0.310 | -0.259 | -0.629 |
|  |  | p value | 0.820 | 0.753 | 0.646 | 0.733 | 0.770 | 0.362 |
|  | **VN RTs** | R | -0.457 | -0.371 | -0.241 | -0.475 | -0.247 | -0.777 |
|  |  | p value | 0.820 | 0.753 | 0.646 | 0.733 | 0.770 | 0.276 |
| **HC** | **NV accuracy** | R | 0.027 | -0.354 | -0.113 | 0.167 | -0.298 | -0.259 |
|  |  | p value | 0.920 | 0.468 | 0.903 | 0.641 | 0.987 | 0.871 |
|  | **VN accuracy** | R | -0.056 | -0.250 | -0.373 | 0.134 | -0.022 | -0.154 |
|  |  | p value | 0.920 | 0.468 | 0.616 | 0.641 | 0.987 | 0.871 |
|  | **NV RTs** | R | 0.165 | 0.268 | 0.141 | 0.217 | 0.116 | 0.122 |
|  |  | p value | 0.920 | 0.468 | 0.903 | 0.641 | 0.987 | 0.871 |
|  | **VN RTs** | R | 0.035 | 0.120 | 0.025 | 0.126 | 0.004 | -0.012 |
|  |  | p value | 0.920 | 0.658 | 0.927 | 0.641 | 0.987 | 0.966 |
